# Multi-Dimensional and Objective Assessment of Motion Sickness Susceptibility Based on Machine Learning

**DOI:** 10.3389/fneur.2022.824670

**Published:** 2022-04-01

**Authors:** Cong-cong Li, Zhuo-ru Zhang, Yu-hui Liu, Tao Zhang, Xu-tao Zhang, Han Wang, Xiao-cheng Wang

**Affiliations:** ^1^Center of Clinical Aerospace Medicine, School of Aerospace Medicine, Fourth Military Medical University, Xi'an, China; ^2^Department of Aviation Medicine, The First Affiliated Hospital, Fourth Military Medical University, Xi'an, China; ^3^Department of Pathophysiology, Medical College, Yan'an University, Yan'an, China; ^4^Department of Medical Electronic Engineering, School of Biomedical Engineering, Fourth Military Medical University, Xi'an, China

**Keywords:** motion sickness, susceptibility, objective assessment, machine learning, vestibular system

## Abstract

**Background:**

As human transportation, recreation, and production methods change, the impact of motion sickness (MS) on humans is becoming more prominent. The susceptibility of people to MS can be accurately assessed, which will allow ordinary people to choose comfortable transportation and entertainment and prevent people susceptible to MS from entering provocative environments. This is valuable for maintaining public health and the safety of tasks.

**Objective:**

To develop an objective multi-dimensional MS susceptibility assessment model based on physiological indicators that objectively reflect the severity of MS and provide a reference for improving the existing MS susceptibility assessment methods.

**Methods:**

MS was induced in 51 participants using the Coriolis acceleration stimulation. Some portable equipment were used to digitize the typical clinical manifestations of MS and explore the correlations between them and Graybiel's diagnostic criteria. Based on significant objective parameters and selected machine learning (ML) algorithms, several MS susceptibility assessment models were developed, and their performances were compared.

**Results:**

Gastric electrical activity, facial skin color, skin temperature, and nystagmus are related to the severity of MS. Among the ML assessment models based on these variables, the support vector machine classifier had the best performance with an accuracy of 88.24%, sensitivity of 91.43%, and specificity of 81.25%.

**Conclusion:**

The severity of symptoms and signs of MS can be objectively quantified using some indicators. Multi-dimensional and objective assessment models for MS susceptibility based on ML can be successfully established.

## Introduction

Motion sickness (MS), as a response to exposure to a provocative environment, cannot essentially be called a disease. However, with the extension of activities from land and sea to the sky and outer space, the transformation of information sources from paper-based text and pictures to digital multimedia resources, and the increasing replacement of work by intelligent machines, MS has a greater effect on humans. MS has also evolved into several types, such as carsickness, seasickness, airsickness, space motion sickness, and visually induced motion sickness (VIMS), and there are not a few people who have experienced it (1–3). MS usually manifests as nausea, vomiting, pallor, cold sweat, drowsiness, headache, dizziness, and nystagmus; “Sopite syndrome” (4) and “Mal de debarquement syndrome (MdDS)” (5) are its special manifestations. At present, the pathogenesis of MS has not been fully elucidated, but the “sensory conflict theory,” which revolves around the vestibular nervous system, is the most widely accepted (6). Research and exploration of the central mechanism proposed by this theory are ongoing (7).

Some scholars are keen to study why MS occurs, while others are more concerned with who is susceptible to MS and unable to work normally in provocative environments. Preventing the entry of people susceptible to MS into navigation, aviation, and aerospace occupations is important for maintaining the safety of tasks and the health of personnel. The assessment methods for the susceptibility to MS include tests in the actual environment, provocative tests, medical history surveys, psychological tendency tests, physiological tendency tests, and adaptation observations. Among them, provocative tests with a relatively high economy, safety, and accuracy are frequently used. Subjective rating scales for the severity of symptoms and signs of MS in participants have often been used in previous provocative tests; they include Graybiel's rating scales (8), Wiker's rating scales (9), and simulator sickness questionnaire (SSQ) for VIMS (10). These subjective evaluation methods are easy and convenient, but their accuracy is easily influenced by the motivation of the participant and the experience of the tester.

Hence, several scholars have begun research into various objective evaluation methods and indicators to assess the susceptibility of people to MS. Temperature (TEMP) (11), skin conductance level (SCL) (12), electrogastrography (EGG) (13), and heart rate variability (14) obtained solely from body surface recordings have a long history as objective indicators for assessing MS susceptibility, and Gavgani et al. found that SCL in the forehead was the best physiological correlate of VIMS-induced nausea symptoms (15). Objective assessments of MS susceptibility based on the results of vestibular function tests, such as the vestibulo-ocular reflex for different vestibular receptors (16–18), vestibular evoked myogenic potentials (19–21), and computerized dynamic posturography (22), are gradually becoming the focus of research, among them, the gain asymmetry of the video head impulse test, the nystagmus slow-phase velocity evoked by the caloric test and the amplitude of the cervical vestibular evoked myogenic potential vary in groups of participants with different susceptibility to MS. Functional brain assessments, such as electroencephalogram (23, 24), functional magnetic resonance imaging (25, 26) and functional near-infrared spectroscopy (27, 28), have facilitated the objective assessment of MS susceptibility at the level of higher nerve centers, meanwhile, the correlation between the intensity of activity in certain brain regions during MS exposure and the degree of MS discomfort have been confirmed by several studies. In addition, the levels of arginine vasopressin, ghrelin and immunoglobulins in blood after MS exposure have been found to correlate with the severity of MS (29, 30), while the discovery of single-nucleotide polymorphism and chromosomes associated with MS susceptibility provides new alternatives for the assessment of MS susceptibility (31, 32). However, some of the methods and indicators mentioned above are not suitable for screening large populations, either because the equipment is not portable, or the testing environments are demanding, or invasive manipulations are required to obtain samples.

Thus, the aim of this study was to induce MS using a simple provocative stimulus and objectively quantify its typical manifestations of nausea, vomiting, pallor, cold sweat, dizziness, and nystagmus using portable instruments to explore the correlations between them and MS severity. Using significant objective parameters and several machine learning (ML) algorithms, multi-dimensional and objective MS susceptibility assessment models were developed, and their performances were compared to provide a reference for improving the existing MS susceptibility assessment methods.

## Materials and Methods

### Participants

In order to obtain objective indicators related to the severity of MS, sample size estimation for Pearson correlation analyses were performed using PASS 15 (NCSS Statistical Software, USA), with a threshold of *p* = 0.05, a power of 0.9, and a predicted correlation coefficient between 0.4 and 0.6, yielding a sample size N between 24 and 61. The trial recruited 51 male participants (mean age, 24.54 ± 3.19 years; mean height 175.25 ± 5.80 cm; mean weight, 71.54 ± 2.89 kg), and none of them had a history of epilepsy, increased intracranial pressure, vertigo, cerebrovascular accident, severe mental illness, and drug abuse. None of the participants took any medications or alcoholic beverages within 48 h, and they participated in the experiment 1 h after eating. Physical examination of each participant showed no signs of external otitis, tympanic membrane perforation, or spontaneous nystagmus. Their naked or corrected visual acuity were 1.0 at least and color vision were normal. The study was approved by the Ethics Committee of the First Affiliated Hospital of the Fourth Military Medical University (number: KY-20202054-F-2). Each participant signed an informed consent form and volunteered to participate.

### Equipment and Materials

An electric rotating chair (VTS-0, Peace, China) and Coriolis acceleration stimulation were used to induce MS. The videonystagmography (VNG) equipment (YD-III, Isen, China) fixed on the back of the electric rotating chair was used to record the nystagmus of each participant after the electric rotating chair stopped suddenly. The wireless physiological signal acquisition and analysis system (PhysioLab, Ergoneers, Germany) has multiple sensor channels, such as those for TEMP, SCL, and EGG, and can display and record data in real-time on a computer via Bluetooth. A portable colorimeter (TS7600, 3 nh, China) can be held by one hand to collect skin color parameters, and the skin color can be digitized through the CIE-L^*^a^*^b^*^ color system (33). A posturography equipment (Balance-A, Nuocheng, China) was located next to the electric rotating chair and can be used to quickly assess the equilibrium of the participant. Graybiel's rating scale was used to assess the severity of MS in participants.

### Procedure

**Figure 1:**
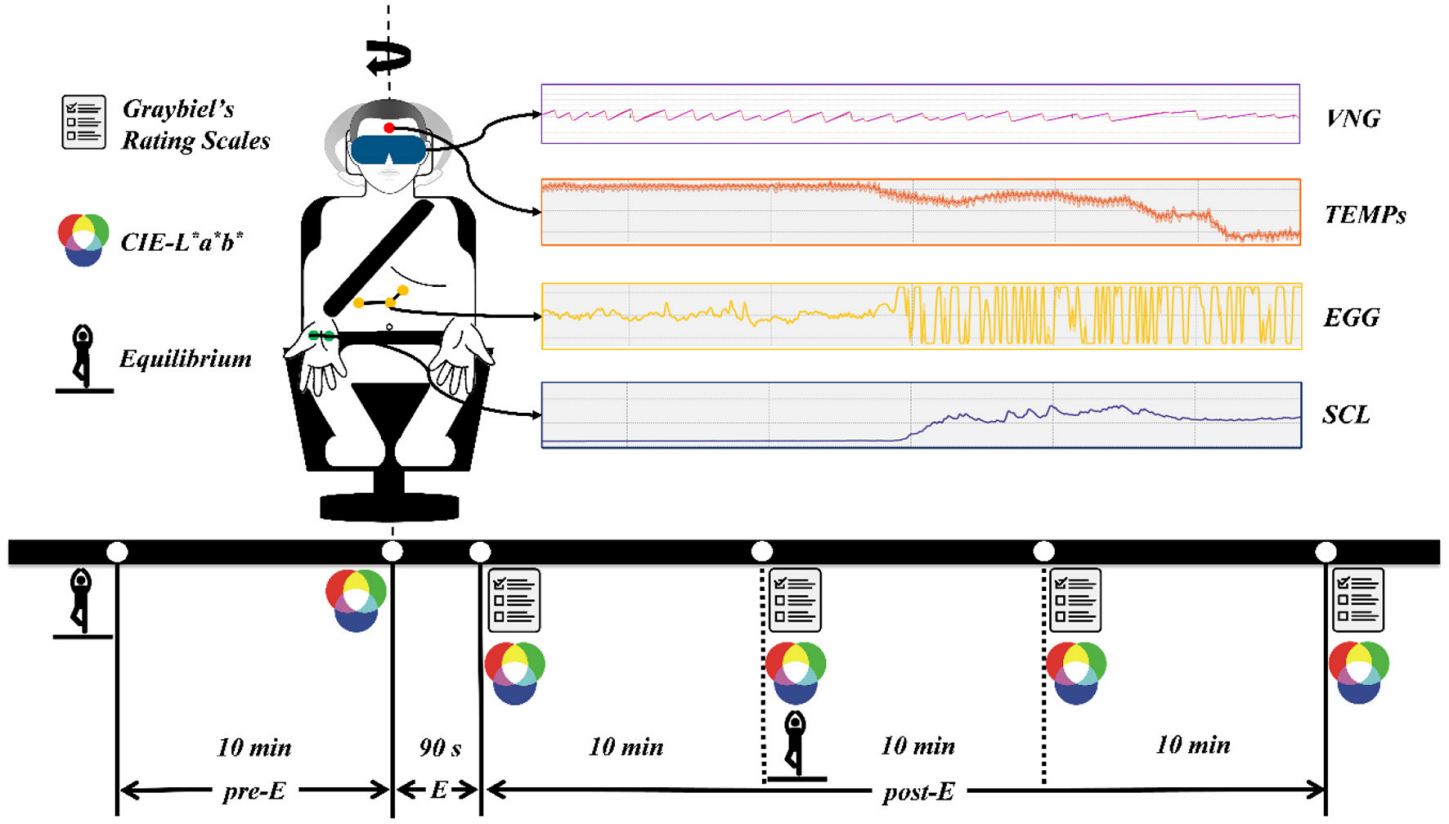
The experimental procedure and data acquisition methods. pre-E, the period before exposure to Coriolis acceleration stimulation; E, the period of exposure to Coriolis acceleration stimulation; post-E, the period after exposure to Coriolis acceleration stimulation.

All tests were carried out in a laboratory with no airflow but automatic light adjustment, and the room temperature was between 20 and 26°C. After reaching the laboratory, each participant took a 30-min break to acclimatize to the test environment. During this period, the participants were shown the movements that produced the Coriolis acceleration stimulation and were requested to truthfully describe their subjective feelings during the test. Considering the late-onset effect of the vestibulo-autonomic response, the entire test was divided into three phases: 10 min for Pre-exposure (pre-E), 90 s of exposure (E), and 30 min for Post-exposure (post-E) to Coriolis acceleration stimulation (Figure 1). The MS of the participants was induced by sitting on a rotating chair at an angular speed of 180°/s for 90 s, during which their heads were swung from a 30° leftward tilt to a 30° rightward tilt (from a 30° rightward tilt to a 30° leftward tilt) once every 2 s.

### Data Acquisition and Processing

Experimental data were obtained by various instruments through continuous recording or acquisition at specific time points, and the data acquisition methods are presented in Figure 1. Skin temperature (TEMP_S_), SCL, and EGG signals were recorded continuously at a sampling frequency of 1 kHz throughout the experiment. VNG data were collected at a sampling frequency of 30 Hz after the emergency stopping of the rotating chair and with the eyes of the participant open. CIE-L^*^a^*^b^*^ data were obtained by measuring the skin color of the left and right cheeks of the participant, and five measurements were taken during the pre- and post-E periods (Figure 1). The equilibrium data were collected with the eyes of the participant closed 10 min before and 10 min after exposure to Coriolis acceleration stimulation. Graybiel's rating scales were used four times during the post-E period (Figure 1), and the severity of MS was assessed for seven components, including nausea syndrome, skin color, cold sweat, salivation, drowsiness, pain, and central nervous system abnormalities.

TEMPs and SCL were obtained from PhysioLab 2021 (Ergoneers, Germany) for exact values. The EGG of nausea and vomiting in MS is mainly characterized by 4–10 cycles per min (cpm) of stomach electrical activity increase (34), and this irregular activity was defined as tachyarrhythmia. Therefore, for the EGG signal, we applied a high-pass filter with a cut-off frequency of 0.067 Hz and a low-pass filter with a cut-off frequency of 0.167 Hz using EDF browser 1.56 (open-source software) and performed a frequency domain analysis of the 0.067–0.167 Hz band to obtain the amplitude (AMP_EGG0.067−0.167_) and power (POW_EGG0.067−0.167_) of the EGG signal in this band. We used the ISEN-VNG 2.1 (ISEN, China) to select three maximal slow-phase velocities (MSPVs) for each VNG, and their average value (MSPV_L−R_) was used for analysis. In the CIE-L^*^a^*^b^*^ color system, the CIE-L^*^ value indicates that the color changes from black to white, the CIE-a^*^ value indicates that the color changes from green to red, and the CIE-b^*^ value indicates that the color changes from blue to yellow (33). Hence, CIE-L^*^ and CIE-a^*^ values were chosen to assess the pallor at the onset of MS and were analyzed using the average of the left and right cheek measurements (CIE-L^*^_L−R_, CIE-a^*^_L−R_). The equilibrium of the participants was assessed primarily by measuring their total traveled way (TTW) and envelope area (EA) of the center of pressure. Graybiel's rating scales were applied by the same senior otolaryngologist, and the sum of the maximum values of the seven dimensions of these rating scales at each specific time point was obtained as Graybiel's score_max_.

### Statistical Analysis

The statistical analyses of the data in this study had the following three steps. (1) First, objective indicators that showed significant changes before and after exposure to MS were determined. The AMP_EGG0.067−0.167_ and POW_EGG0.067−0.167_ data were taken as the baseline for 10 min of the pre-E period and as control for the first 10 min of the post-E period. The TEMPs, SCL, CIE-L^*^_L−R_, and CIE-a^*^_L−R_ were compared using the extreme values measured before and after the Coriolis acceleration stimulation. The TTW and EA were compared before and after the Coriolis acceleration stimulation. A paired *t*-test was used to analyze the differences in each objective indicator. (2) Second, objective indicators that changed significantly before and after exposure to MS and whose variations correlated with the severity of MS were screened. Two-tailed Pearson's correlation analyses were carried out for the change in the objective indicators selected in step 1 and the Graybiel's score_max_. MSPV_L−R_ was also analyzed for Pearson's correlation with the Graybiel's score_max_. (3) Several assessment models of MS susceptibility based on ML were established. We used the variations of the objective indicators selected in step 2 as input variables; “Graybiel's score_max_ ≥ 5” as the output indicating “susceptible to MS” and “Graybiel's score_max_ <5” as the output indicating “not susceptible to MS” (35), to develop MS susceptibility assessment models by using different ML algorithms. We also compared the performances of the models. Statistical analyses and charting were performed using SPSS 22.0 (IBM Corporation, USA), GraphPad Prism 8.0 (GraphPad Software, USA), and Origin 2018 (OriginLab Corporation, USA). Statistical significance was set at *p* < 0.05.

### ML Algorithms and Tools

In this study, four widely applied ML algorithms, including support vector machine (SVM), random forest (RF), K-nearest neighbor (KNN), and multilayer perceptron (MLP), were used to build the assessment models of MS susceptibility. SVM separates different types of data points by drawing a virtual hyperplane and is widely used for small sample data (36). RF is an integrated classifier consisting of several randomly generated decision tree classifiers, which has a better classification performance than traditional decision tree algorithms (37). KNN finds the k-nearest training samples to the test samples based on a distance measure and uses the information of these k “neighbors” to classify the test samples (38). As a feedforward artificial neural network, MLP can be used to construct effective classifier algorithms to distinguish Non-linear separable data (39).

We used the Scikit-learn ML algorithm package based on Python 3.7 to build the above four classifiers (40), and the performances of these models were compared. On the one hand, we selected 70% of the 51 samples as the training set and 30% as the test set for dichotomous classification of MS susceptibility, and we performed a stratified random sampling according to the proportion of participants “susceptible to MS” and “not susceptible to MS” when selecting data for the training and test sets. The receiver operator characteristic curve (ROC) and area under the ROC (AUC) of each classifier were obtained. The above processes were repeated 100 times to obtain the respective average AUCs of the four models. In contrast, a 10-fold cross-validation was performed on the 51 samples to compare the accuracy, sensitivity, specificity, positive predictive value (precision), and negative predictive value of the four models.

## Results

All 51 participants successfully completed the experiment following the procedure and honestly reported various physical discomforts at different time points after exposure to MS. After processing and statistical analyses of all experimental data, the following results were obtained.

### Effects of the Coriolis Acceleration Stimulation on Objective Indicators

**Figure 2:**
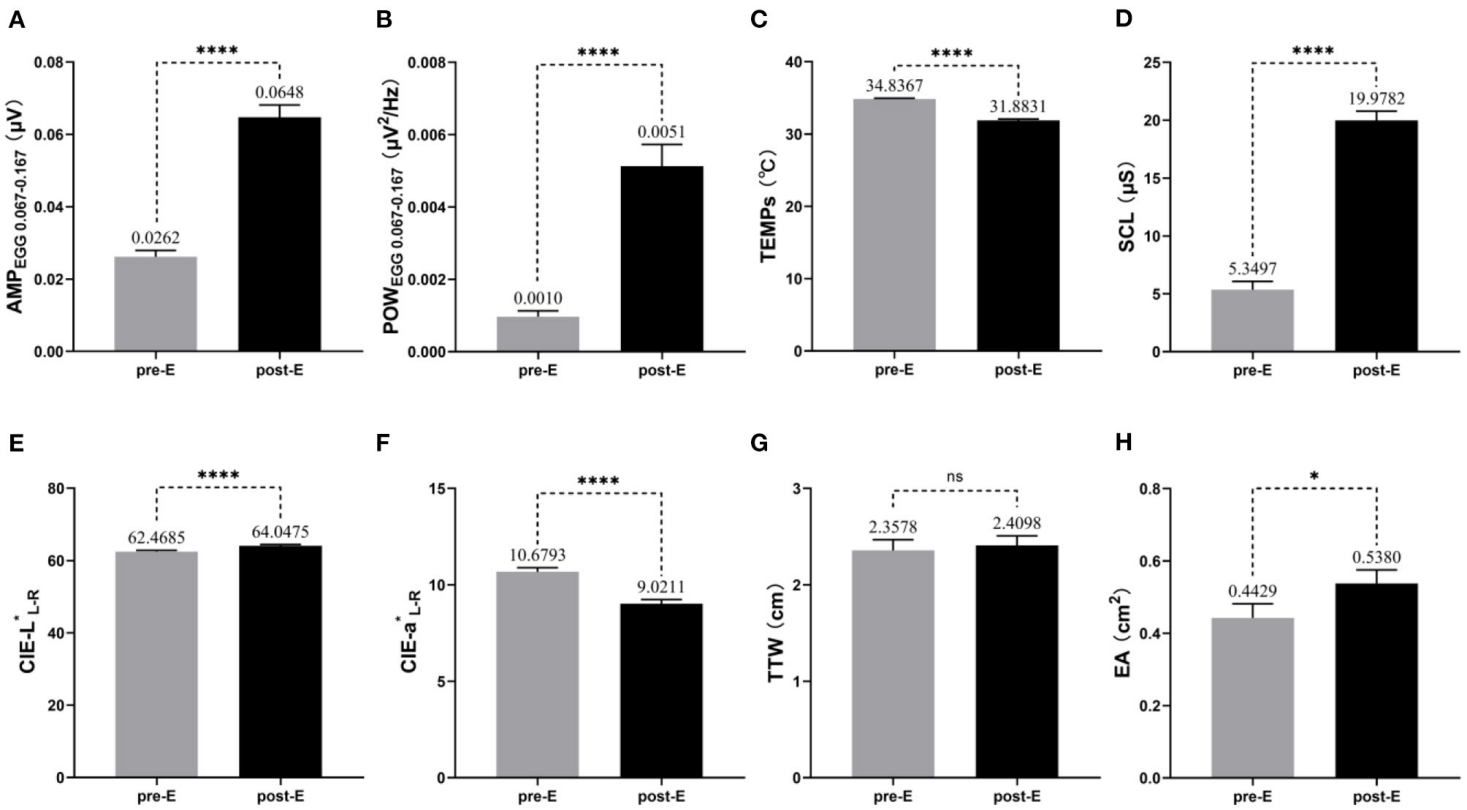
Changes in objective indicators before and after exposure to MS (*n* = 51). The mean and standard error of measured values for each objective indicator during the pre-E and post-E periods are also labeled in the pictures. **(A)** AMP_EGG0.067−0.167_; **(B)** POW_EGG0.067−0.167_; **(C)** TEMPs; **(D)** SCL; **(E)** CIE-L^*^_L−R_; **(F)** CIE-a^*^_L−R_; **(G)** TTW; **(H)** EA. * *p* < 0.05; ****p < 0.0001; ns, no significance.

As shown in Figure 2, compared to the baseline before exposure, most of the objective indicators we focused on changed visually after exposure to Coriolis acceleration stimulation. Paired *t*-tests showed that AMP_EGG0.067−0.167_, POW_EGG0.067−0.167_, SCL, CIE-L^*^_L−R_, and EA increased significantly (*t* = 12.27, *p* < 0.0001 for AMP_EGG0.067−0.167_, Figure 2A; *t* = 7.151, *p* < 0.0001 for POW_EGG0.067−0.167_, Figure 2B; *t* = 17.77, *p* < 0.0001 for SCL, Figure 2D; *t* = 12.05, *p* < 0.0001 for CIE-L^*^_L−R_, Figure 2E; *t* = 2.451, *p* = 0.0178 for EA, Figure 2H), while TEMPs and CIE-a^*^_L−R_ decreased significantly (*t* = 17.02, *p* < 0.0001 for TEMPs, Figure 2C; *t* = 14.03, *p* < 0.0001 for CIE-a^*^_L−R_, Figure 2F), but TTW did not change remarkably (*t* = 0.6095, *p* = 0.5449, Figure 2G). The trend of changes in the above objective indicators was consistent with the typical signs and symptoms of MS.

### Objective Indicators That Can Reflect the Severity of MS

**Figure 3:**
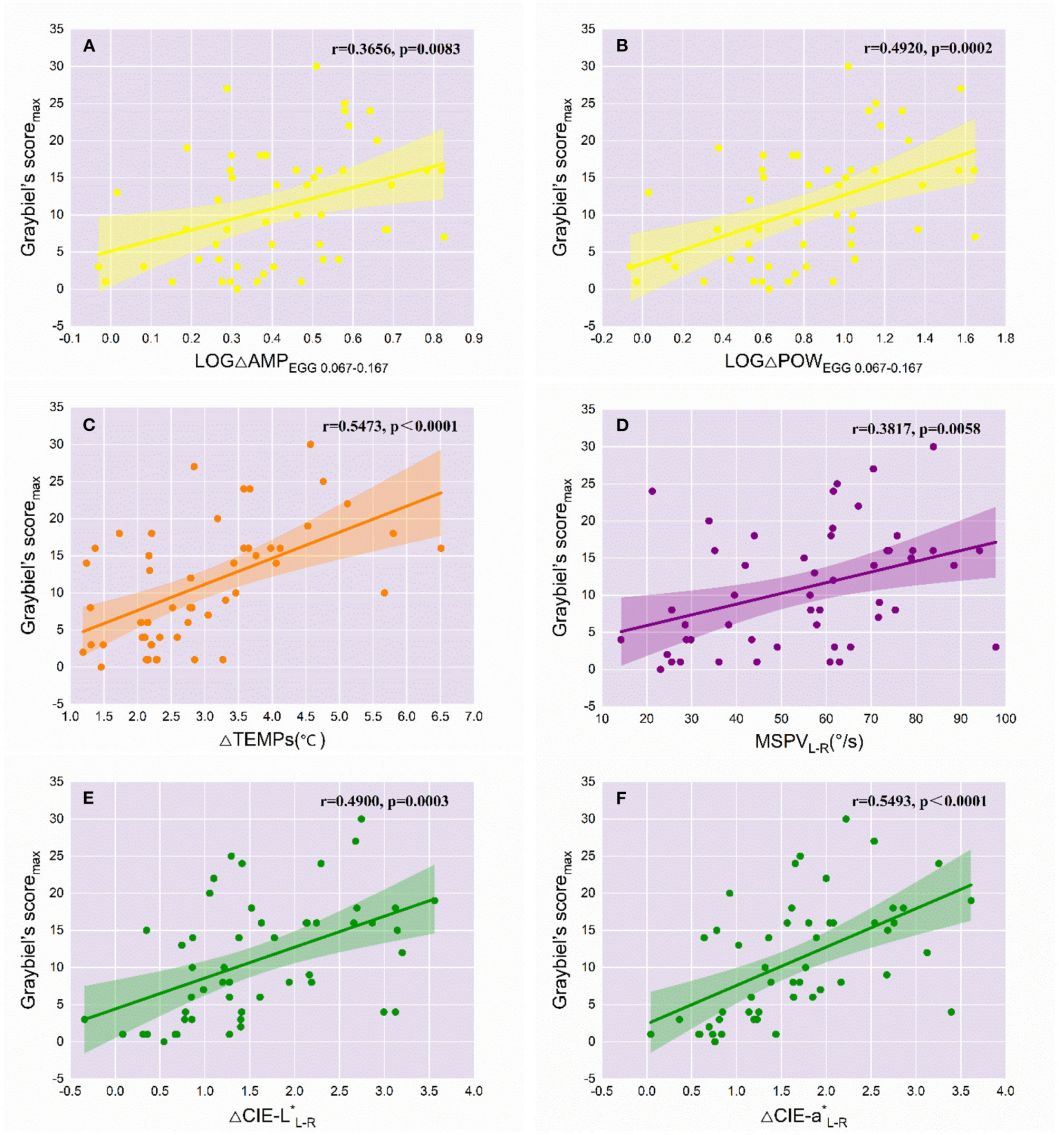
Correlations of the variations of objective indicators with the severity of MS (*n* = 51). The *r* and *p* values of the objective variables significantly correlated with Graybiel's score_max_ are labeled in each graph. The colored line represents the line of best fit for each linear regression, while the shaded area indicates the 95% confidence interval. **(A)** LOGΔAMP_EGG0.067−0.167_; **(B)** LOGΔPOW_EGG0.067−0.167_; **(C)** ΔTEMPs; **(D)** MSPV_L−R_; **(E)** ΔCIE-L^*^_L−R_; **(F)** ΔCIE-a^*^_L−R_.

Because TTW did not change significantly before and after Coriolis acceleration stimulation, it was not included in the correlation analyses with Graybiel's score_max_, which represents the severity of MS. Given the exponential increase in the EGG-related indicators, we obtained the logarithmic values of multiples of change in AMPEGG _0.067−0.167_ and POW_EGG0.067−0.167_ (LOGΔAMP_EGG0.067−0.167_ and LOGΔPOW_EGG0.067−0.167_), with the change in the values of other meaningful objective indicators and MSPV_L−R_, for Pearson's correlation analyses with Graybiel's score_max_. For LOGΔAMP_EGG0.067−0.167_ (Figure 3A), the correlation with Graybiel's score_max_ was significant (*r* = 0.3656, *p* = 0.0083). Also significantly correlated with Graybiel's score_max_ were LOGΔPOW_EGG0.067−0.167_ (*r* = 0.4920, *p* = 0.0002, Figure 3B), ΔTEMPs (*r* = 0.5473, *p* < 0.0001, Figure 3C), MSPV_L−R_ (*r* = 0.3817, *p* = 0.0058, Figure 3D), ΔCIE-L^*^_L−R_ (*r* = 0.4900, *p* = 0.0003, Figure 3E), and ΔCIE-a^*^_L−R_ (*r* = 0.5493, *p* < 0.0001, Figure 3F). As a result, the above 6 variables could objectively represent the severity of MS in some degree. However, no significant correlation was found between ΔSCL and Graybiel's score_max_ (*r* = 0.2009, *p* = 0.1574), as well as ΔEA (*r* = −0.1392, *p* = 0.3302).

### ML Classifiers and Their Performance Comparison

**Figure 4:**
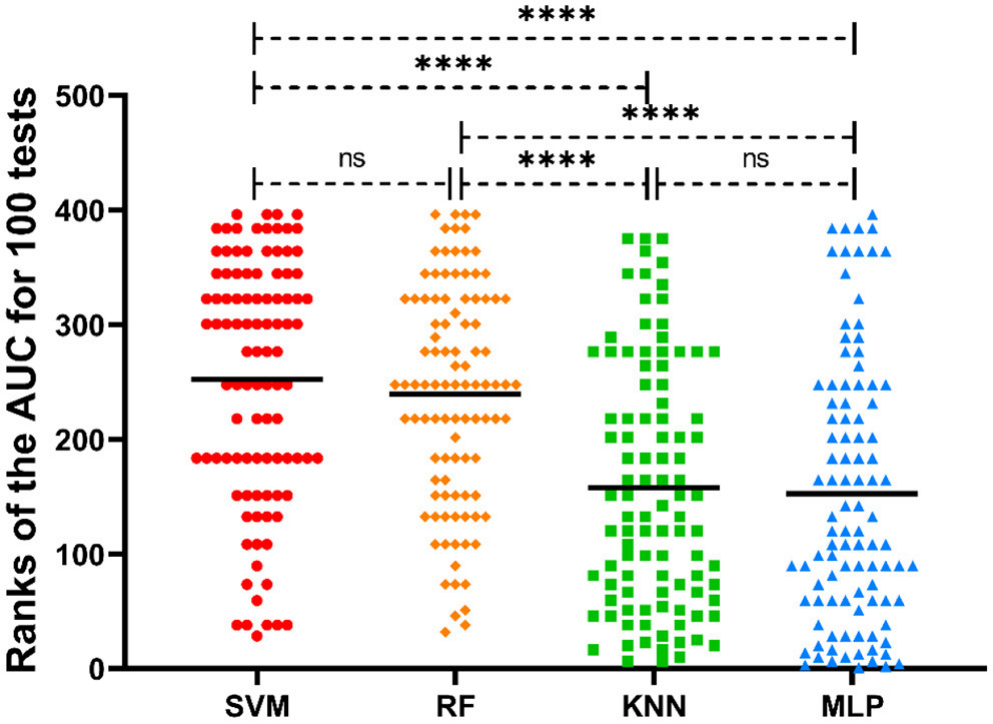
Distribution of the ranks of AUC values of different ML classifiers for 100 tests. The mean ranks of the AUC values for each model are also labeled in the picture. ****p < 0.0001; ns, no significance.

The SVM, RF, KNN, and MLP classifiers for MS susceptibility were based on the above 6 variables related to the severity of MS. The means and standard deviations of the AUC values of the four models were obtained by training and testing with 100 stratified random samples (Table 1), and the difference between the AUC values of the groups was confirmed by the Kruskal-Wallis test and Dunn's multiple comparisons test (Figure 4). Among the four models, the SVM classifier had the best performance, with the highest average AUC value (0.8795 ± 0.0815) for 100 times of classification training and testing.

**Table 1:** Means and standard deviations of AUC for 100 tests of the ML classifier.

**Classifier**	**Mean**	**Standard deviation**
SVM	**0.8795**	**0.0815**
RF	0.8703	0.0716
KNN	0.8007	0.0925
MLP	0.7904	0.1113

*The bold values indicate that the SVM classifier had the best performance among the four models*.

**Figure 5:**
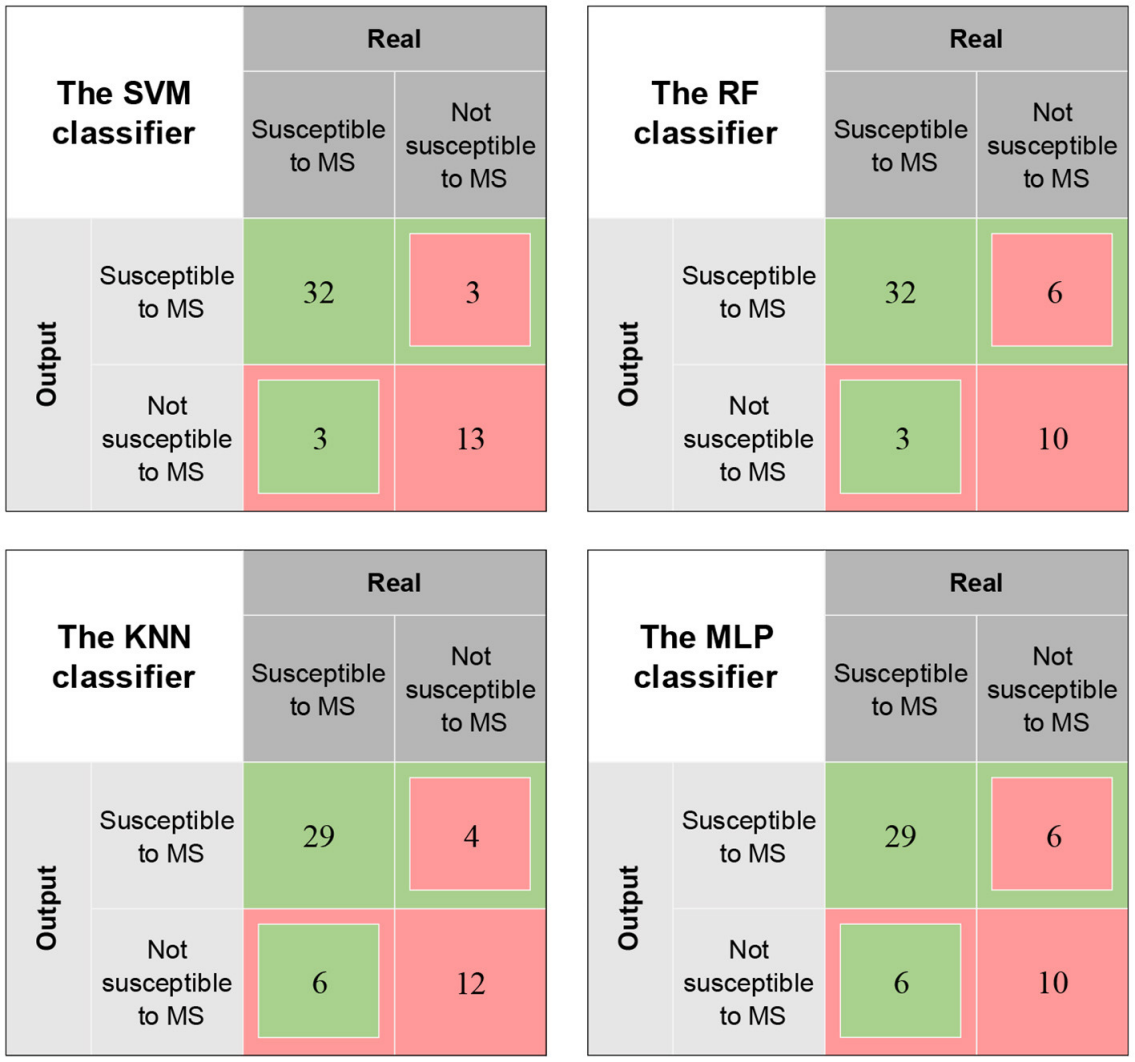
Confusion matrix for the classification results of different classifiers.

**Figure 6:**
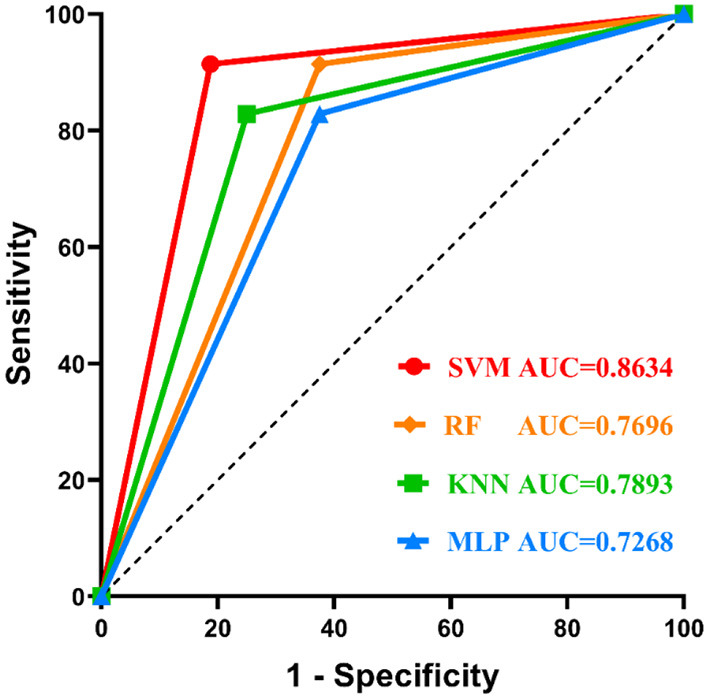
Comparison of the ROC curves of different ML classifiers. The SVM classifier performs best.

We also presented the classification results of the 10-fold cross-validation of the four models for 51 samples using a confusion matrix (Figure 5) and compared the performances of the ML classifiers (Table 2 and Figure 6). As can be seen, also, SVM classifier performed well with an accuracy of 88.24%, sensitivity of 91.43%, specificity of 81.25% and AUC value of 0.8634 for predicting MS susceptibility. In general, the classification results of SVM classifier were the most satisfactory.

**Table 2:** Evaluation of the performances of different ML classifiers.

**Classifier**	**Accuracy**	**Sensitivity**	**Specificity**	**Positive predictive value (precision)**	**Negative predictive value**
SVM	**0.8824**	**0.9143**	**0.8125**	**0.9143**	**0.8125**
RF	0.8236	0.9143	0.6250	0.8421	0.7692
KNN	0.8039	0.8286	0.7500	0.8789	0.6667
MLP	0.7647	0.8286	0.6250	0.8286	0.6250

*The bold values indicate that the SVM classifier had the best performance among the four models*.

## Discussion

For the general public, an objective and accurate understanding of their susceptibility to MS can help them choose more comfortable means of transportation and recreation and actively prevent the adverse effects of MS. However, for people working in special environments, such as drivers, astronauts, pilots, sailors, divers, extreme sports personnel, and operators of unmanned equipment or simulators, exposure to provocative motion environments is usually inevitable. Therefore, it is worthwhile to reduce the influence of human subjective factors and implement an objective and accurate assessment of the susceptibility to MS in those who are ready to join the above-mentioned special jobs.

Our study induced MS with the normative Coriolis acceleration stimulus and objectively quantified the typical manifestations of MS, such as nausea, vomiting, pallor, cold sweat, dizziness, and nystagmus, using a reproducible approach. We successfully sieved out several parameters related to gastric electrical activity, facial skin color, skin temperature, and nystagmus that could objectively assess the severity of MS, and these findings are similar to the results of previous studies. Chinese scholars also used Coriolis acceleration stimulation to induce MS and found that the tachyarrhythmia of EGG was significantly enhanced in participants with severe MS (41), but they only compared EEG differences between groups with different levels of MS; Gruden et al. used a vehicle simulator to induce VIMS and found that the magnitude and duration of elevated EGG signals in participants correlated with their subjective level of nausea (42), but they used the SSQ scales, which are more suitable for VIMS, as the reference for correlation analyses. It has been reported that the magnitude of reduction in TEMP correlates with the degree of subjective discomfort induced by different levels of MS (43), a study on the treatment of MS consistently showed the efficacy of “promethazine + dextroamphetamine” in reducing MS symptoms and preventing a core temperature drop (44), but these studies also only compared the magnitude of TEMP decrease between groups with different levels of MS or with different anti-MS drugs. Both the MSPV of vestibulo-ocular reflex induced by the caloric test and the rotating chair test have been proven to be associated with susceptibility to MS (18, 45), however, neither of the related studies was grouped by severity of MS after the Coriolis acceleration stimulation. By contrast, our selected objective indicators better achieved a linear quantitative assessment of the severity of MS. In particular, to the best of our knowledge, our study is the first to assess the severity of MS by objectively measuring skin color.

Meanwhile, we did not find a significant correlation between the severity of MS and the changes in SCL, TTW, and EA, which is not consistent with the reports of previous studies. When Gavgani et al. studied VIMS, they found that SCL of the fingers was significantly correlated with the degree of nausea, but they only classified nausea as one symptom of MS into four levels to analyze the data (15), unlike in the current study that correlated the changes in skin SCL with the exact scores of Graybiel's rating scales, and the difference between the two results was acceptable. Moreover, various physiological and psychological changes such as excitement, anger, fear, agitation, and fatigue can cause fluctuations in skin SCL, which is also sensitive to variations in external elements such as temperature, humidity, air flow, and clothing; therefore, it is challenging to objectively and accurately assess the severity of MS using SCL as the only indicator. In addition, the possible reasons for the insignificant correlations between the changes in TTW and EA and the severity of MS in this study are as follows. (1) First, to prevent the effect of body movement on the accuracy of other physiological parameters collected, we performed the second equilibrium test 10 min after the Coriolis acceleration stimulation; the long period for recovery may have mildened the symptoms of dizziness and instability in MS susceptible persons. (2) Since the participants were not familiarized with the method for equilibrium measurement before the test, the training effect may have also affected the test results. Further studies are needed to verify whether optimizing the experimental design will result in new findings.

Previous studies have focused on the screening of physiological markers that can objectively evaluate MS and VIMS, and ML modeling for MS susceptibility assessment has mostly targeted VIMS (25, 46, 47). In this study, we induced MS with real vestibular stimuli, as well as pioneered the collection of objective physiological parameters from multiple dimensions simultaneously, and the use of various ML methods to establish the optimal model for MS susceptibility assessment. According to the experimental results, the assessment model based on the SVM was optimal because of its relatively high sensitivity and specificity, which facilitates not only the identification of people susceptible to MS but also, and more importantly, the selection of people who are not susceptible to MS for special jobs where they are frequently exposed to provocative motion environments.

The pathogenesis of MS has not yet been fully elucidated. However, according to the classical “sensory conflict theory” of MS, all types of sensory conflicts that cause MS do not lack the information input from the vestibular system (6). The vestibular nuclei, as a repeater of vestibular information input and efferent, have extensive fiber connections with vision, proprioception, and other brain nuclei (48), and the susceptibility to MS is reduced in those with vestibular loss or hypofunction (49). Thus, it is reasonable to believe that the functional state of the vestibular system plays a crucial role in the onset and development of MS, and many clinical manifestations of MS are exported through different vestibular pathways. Based on this, we selected objective indicators that can be easily quantified in the vestibular pathway for the experiment, such as EGG, TEMPs, SCL, CIE-L^*^, and CIE-a^*^ in the vestibulo-autonomic pathway, MSPV in the vestibulo-ocular pathway, and TTW and EA in the vestibulo-spinal pathway. We were pleased to find that participants with a mild MS response to the same intensity of provocative vestibular stimulus showed relatively small changes in the magnitude of the objective indicators, and the subjective severity of MS correlated well with the objective output based on the vestibular pathways. However, people with mild MS are able to maintain body balance and perform spatial orientation normally, just as their vestibular system is stable and does not overreact to external provocative stimuli. This is comparable to the normal immune system, which can resist pathogens and maintain health without overly reacting to antigens and causing allergic reactions or autoimmune diseases. From this perspective, the assessment of MS susceptibility is similar to performing a skin (or intradermal) sensitivity test, but the skin (or intradermal) sensitivity test has its own standard operating procedure and objective method for judging the result, which is what we want to do with the assessment of MS susceptibility. Furthermore, with the deepening of basic research on MS, as well as the upgrading of smart wear and artificial intelligence, we hope that our study will provide a reference for the accurate assessment of MS susceptibility, the real-time monitoring of MS, and the development of integrated equipment for “examination-training-assessment” of vestibular function stability to facilitate the prevention and optimal treatment of MS.

## Limitations

Our study has limitations, such as the timing of the acquisition of equilibrium parameters and training effects mentioned in the previous discussion section. It is difficult to precisely regulate temperature and humidity in the laboratory, as well as shield against noise, which may have led to errors in the experimental data. Finally, the sample of our study was not large enough, which may have limited the performance of various ML algorithms for the model and impacted the stability of the evaluation model.

## Conclusion

This study induced MS using a uniform Coriolis acceleration stimulus and obtained some indicators related to gastric electrical activity, facial skin color, skin temperature, and nystagmus, which could be used to objectively and quantitatively assess the severity of MS. Based on these, several assessment models based on different ML algorithms were developed and compared, among which, the SVM model with the best performance could be suitable for a multi-dimensional objective assessment of MS susceptibility.

## Data Availability Statement

The raw data supporting the conclusions of this article will be made available by the authors, without undue reservation.

## Ethics Statement

The studies involving human participants were reviewed and approved by the Ethics Committee of The First Affiliated Hospital of the Fourth Military Medical University. The patients/participants provided their written informed consent to participate in this study.

## Author Contributions

C-cL, Y-hL, X-tZ, and X-cW conceived and designed the experiment methods. C-cL, Z-rZ, Y-hL, and X-cW organized experiments and collected relevant data. C-cL, Z-rZ, TZ, and HW conducted the statistics and machine learning modeling. C-cL, Z-rZ, Y-hL, and TZ drafted the manuscript. X-tZ, HW, and X-cW revised the manuscript. All authors take responsibility for the integrity of the data and the accuracy of data analysis. All authors contributed to the article and approved the submitted version.

## Funding

This work was supported by Medicine Promotion Project (Grant Number 2018HKZL04) and Researcher and Development Plan in Shaanxi (Grant Number 2018SF-252).

## Conflict of Interest

The authors declare that the research was conducted in the absence of any commercial or financial relationships that could be construed as a potential conflict of interest.

## Publisher's Note

All claims expressed in this article are solely those of the authors and do not necessarily represent those of their affiliated organizations, or those of the publisher, the editors and the reviewers. Any product that may be evaluated in this article, or claim that may be made by its manufacturer, is not guaranteed or endorsed by the publisher.
